# Zebra finches (*Taeniopygia guttata*) demonstrate cognitive flexibility in using phonology and sequence of syllables in auditory discrimination

**DOI:** 10.1007/s10071-023-01763-4

**Published:** 2023-03-19

**Authors:** Zhi-Yuan Ning, Henkjan Honing, Carel ten Cate

**Affiliations:** 1 Behavioural Biology, Institute of Biology Leiden, Leiden University, Leiden, The Netherlands; 2 Amsterdam Brain and Cognition, Institute for Logic Language and Computation, University of Amsterdam, Amsterdam, The Netherlands; 3 Leiden Institute for Brain and Cognition, Leiden University, Leiden, The Netherlands

**Keywords:** Syllable sequence, Spectral structure, Auditory perception, Operant discrimination, Birdsong, Zebra finches

## Abstract

Zebra finches rely mainly on syllable phonology rather than on syllable sequence when they discriminate between two songs. However, they can also learn to discriminate two strings containing the same set of syllables by their sequence. How learning about the phonological characteristics of syllables and their sequence relate to each other and to the composition of the stimuli is still an open question. We compared whether and how the zebra finches’ relative sensitivity for syllable phonology and syllable sequence depends on the differences between syllable strings. Two groups of zebra finches were trained in a Go-Left/Go-Right task to discriminate either between two strings in which each string contained a unique set of song syllables (‘Different-syllables group’) or two strings in which both strings contained the same set of syllables, but in a different sequential order (‘Same-syllables group’). We assessed to what extent the birds in the two experimental groups attend to the spectral characteristics and the sequence of the syllables by measuring the responses to test strings consisting of spectral modifications or sequence changes. Our results showed no difference in the number of trials needed to discriminate strings consisting of either different or identical sets of syllables. Both experimental groups attended to changes in spectral features in a similar way, but the group for which both training strings consisted of the same set of syllables responded more strongly to changes in sequence than the group for which the training strings consisted of different sets of syllables. This outcome suggests the presence of an additional learning process to learn about syllable sequence when learning about syllable phonology is not sufficient to discriminate two strings. Our study thus demonstrates that the relative importance of syllable phonology and sequence depends on how these features vary among stimuli. This indicates cognitive flexibility in the acoustic features that songbirds might use in their song recognition.

**Supplementary Information:**

The online version contains supplementary material available at 10.1007/s10071-023-01763-4.

## Introduction

Not only humans, but also songbirds learn their vocalizations early in life from their parents or other individuals. Vocal learning implies the presence of advanced auditory processing, including perception, memorization, and production of complex strings of sounds. Most emphasis in studies of vocal learning and auditory processing in birds is on the processes involved in learning the phonology, i.e., the spectro-temporal structure, of syllables, rather than on learning the syllable sequences (Vernes et al. 2021).

Songbird species show a large diversity in how syllables are arranged within songs. Some songbird species, such as the canary (*Serinus canaria*) (Lehongre et al. 2008), European starling (*Sturnus vulgaris*) (Eens 1997), or willow warbler (*Phylloscopus trochilus*) (Gil and Slater 2000), have a repertoire of syllables that are ordered in varying sequences to form phrases that together make up the song. The sequence of syllables sung within a given song is rarely an exact replicate of the previous song or of a sequence produced by the model from which the syllables are copied. This is in contrast to the vocalizations in species such as the white-crowned sparrow (*Zonotrichia leucophrys*) (Soha and Marler 2001), the chaffinch (*Fringilla coelebs*) (Riebel and Slater 1999), song sparrow (*Melospiza melodia*) (Marler and Peters 1987), or the zebra finch (*Taeniopygia guttata*) (Eales 1985), in which songs consist of rather fixed sequences of syllables, and in which copied songs show limited element sequence divergence from the song models. The fact that these songbirds as well as others faithfully copy both the spectro-temporal structure of song syllables as well as their sequences implies they have the ability to perceive and learn the phonology as well as the sequential order of conspecific syllables in great detail.

The zebra finch is an extensively used model species for comparative studies of vocal learning as well as auditory perception. With respect to sequence learning, despite the fact that zebra finches may have certain non-learned biases as to how different syllable types are distributed over a sequence (James and Sakata 2017), there is ample evidence that syllable sequences are affected by learning (e.g., Eales 1985). This is supported by the finding that zebra finch songs, both in captive and wild populations, show culturally transmitted differences in the position of specific syllable types, being more similar within than between colonies (Lachlan et al. 2016). Also, zebra finches first exposed to one set of syllables in a particular sequence and next exposed to a novel set first acquire the phonological structure of the novel syllables and next adjust the sequence of these novel syllables, indicating the involvement of at least partially different learning processes (Lipkind et al. 2013). Comparable evidence of a separation between learning the phonology of syllables and learning of their sequence can also be found on other songbirds, such as the white-crowned sparrow (e.g., Soha and Marler 2001; Plamondon et al. 2010).

The finding that zebra finches attend to and learn about both phonology and syllable sequence demonstrates that both are perceived and suggests that they are both relevant for communication, for instance to distinguish between individuals. However, experiments addressing which song features zebra finches use to discriminate between songs suggest a striking imbalance between the role of syllable phonology and the role of syllables sequence. For instance, Braaten et al. (2006) used an operant discrimination task (Go/No-go) to train adult and juvenile zebra finches to discriminate the natural forward song from its reversed version (i.e., a song played backwards). Tests in which a song was presented with syllables of non-reversed phonological structure in the reversed sequence and a song in which element sequence was maintained, but the syllables were reversed, showed that the original stimuli were discriminated on the phonological structure of the syllables and not by their sequence. A recent study, also using a Go/No-go task, investigated the role of syllable sequences versus spectro-temporal fine structure of syllables for the process of individual recognition: zebra finches were trained to discriminate songs of one male conspecific from those of four others; thereafter they were exposed to hybrid stimuli combining the syllable sequences of one individual with the spectro-temporal features of another. The results demonstrated that zebra finches mainly rely on spectro-temporal details of syllables and pay less attention to syllable sequences (Geberzahn and Derégnaucourt 2020). A laboratory playback experiment (Mol et al. 2021) also suggested that syllable sequence is not an essential cue for recognition of familiar songs in zebra finches. In another study, Lawson et al. (2018) used a discrimination task to compare the ability of zebra finches to notice changes of syllable phonology and changes of syllable sequence in the motifs of natural songs. These results also showed that zebra finches could readily recognize the reversal of a single syllable in the motif, but largely ignore the change of syllable sequence in the motif. Similarly, zebra finches detect single-syllable reversals more easily than a doubling of an inter-syllable interval (e.g., Dooling and Prior 2017). Combined with evidence that zebra finches can detect differences between renditions of slightly different versions of the same song syllables (Fishbein et al. 2021), demonstrating the attention to fine details of the spectro-temporal structure of syllables, such findings raised the question to what extent zebra finches attend to the sequences of syllables (Fishbein et al. 2019).

Some studies have indicated that syllable sequence can play an additional role in song recognition. Lawson et al. (2018) showed that male zebra finches tested with their own songs or with those of familiar birds attended to sequences of syllables in addition to the spectro-temporal structure of these syllables. So, although zebra finches may thus show a strong bias to attend to spectro-temporal features of syllables to distinguish songs, they can also attend to syllable sequence. It suggests that more extensive experience with songs is needed before the birds acquire knowledge about syllable sequences. This was also suggested by an experiment showing that juvenile zebra finches could discriminate songs on the basis of syllable sequence alone, although this discrimination was more difficult to obtain than one based on syllable structure (Braaten et al. 2006). However, in contrast to the studies indicating a marginal role of syllable sequences in song discrimination and suggesting that learning about sequences might be more difficult than about syllable phonology, a range of studies demonstrated that zebra finches can readily learn to distinguish strings consisting of identical syllables but differing in their sequence (e.g., van Heijningen et al. 2013; Chen and ten Cate 2015, 2017; Chen et al. 2016; Spierings and ten Cate 2016; Knowles et al. 2018). In a study by van Heijningen et al. (2009), zebra finches were trained in a Go/No-go task to discriminate between stimuli in which syllables were arranged in an ABAB or an AABB sequence. They readily acquired this discrimination. When next tested with stimuli of the same sequential structures but constructed of novel exemplars of the same type of syllables (and hence differing in fine spectro-temporal details), they generalized the discrimination to the novel exemplars based on the string structure. Evidence from a neural study (Cazala et al. 2019) also using an AABB vs ABAB paradigm demonstrated that the caudomedial nidopallium (NCM) neurons encode the sequencing of syllables, which also supports the outcome of the behavioral studies described above in showing that zebra finches have no difficulty in distinguishing two strings by the sequence of their syllables. Zebra finches can thus readily use sequence information to distinguish strings differing in their sequence only.

The findings discussed above raise the question how learning about the spectro-temporal characteristics of syllables and about syllable sequences relate to each other and to the composition of the stimuli. The range of experiments mentioned above differ in methods and stimulus composition. So far, no experiment has directly compared the relative importance of spectral structure and sequence when zebra finches have to discriminate two syllable strings that either consist of different sets of syllables or consist of the same set of syllables, but different in the sequence, using similarly structured strings and identical training and testing procedures.

In the current study, we use an operant discrimination paradigm—the Go-Left/Go-Right task—to examine the relative salience of syllable phonology and syllable sequence when zebra finches must distinguish two artificially constructed ‘song motifs’ that are either composed of different syllable types (the ‘Different-syllables group’), or two stimuli composed of the same set of syllables but differing in sequence (the ‘Same-syllables group’). We investigate whether the stimulus contrast in the training affects the ease of learning by examining the speed with which the discrimination is achieved. Next, we assess to what extent the birds in the two groups attend to the syllable phonology by assessing the responses to test strings consisting of reversed syllables or of vocoded versions of these syllables. To examine the importance of syllable sequence, we assessed the responses to test strings in which the sequences are shuffled.

## Methods

### Subjects

Twenty-four zebra finches (12 males, and 12 females; ages 139–691 days post-hatching) were used in this experiment. All birds originated from the in-house breeding colony at Leiden University. Before the experiment, the birds lived in single-sex groups of about 15–30 individuals in aviaries (2 m × 2 m × 1.5 m), in which food and water were available ad libitum.

The birds were divided randomly in two experimental groups; half of the birds were assigned to the Different-syllables group, and the other half of them to the Same-syllables group (6 males and 6 females in each group; age Different-syllables group: M = 309, SD = 184, age Same-syllables group: M = 387, SD = 246). Each group was trained to discriminate between two different strings consisting of five zebra finch syllables. Within each training group one half of the birds got training strings consisting of single-element syllables, and the other half another set of stimuli consisting of one complex syllable and four single-element syllables within a string.

### Operant conditioning cage

**Fig. 1:**
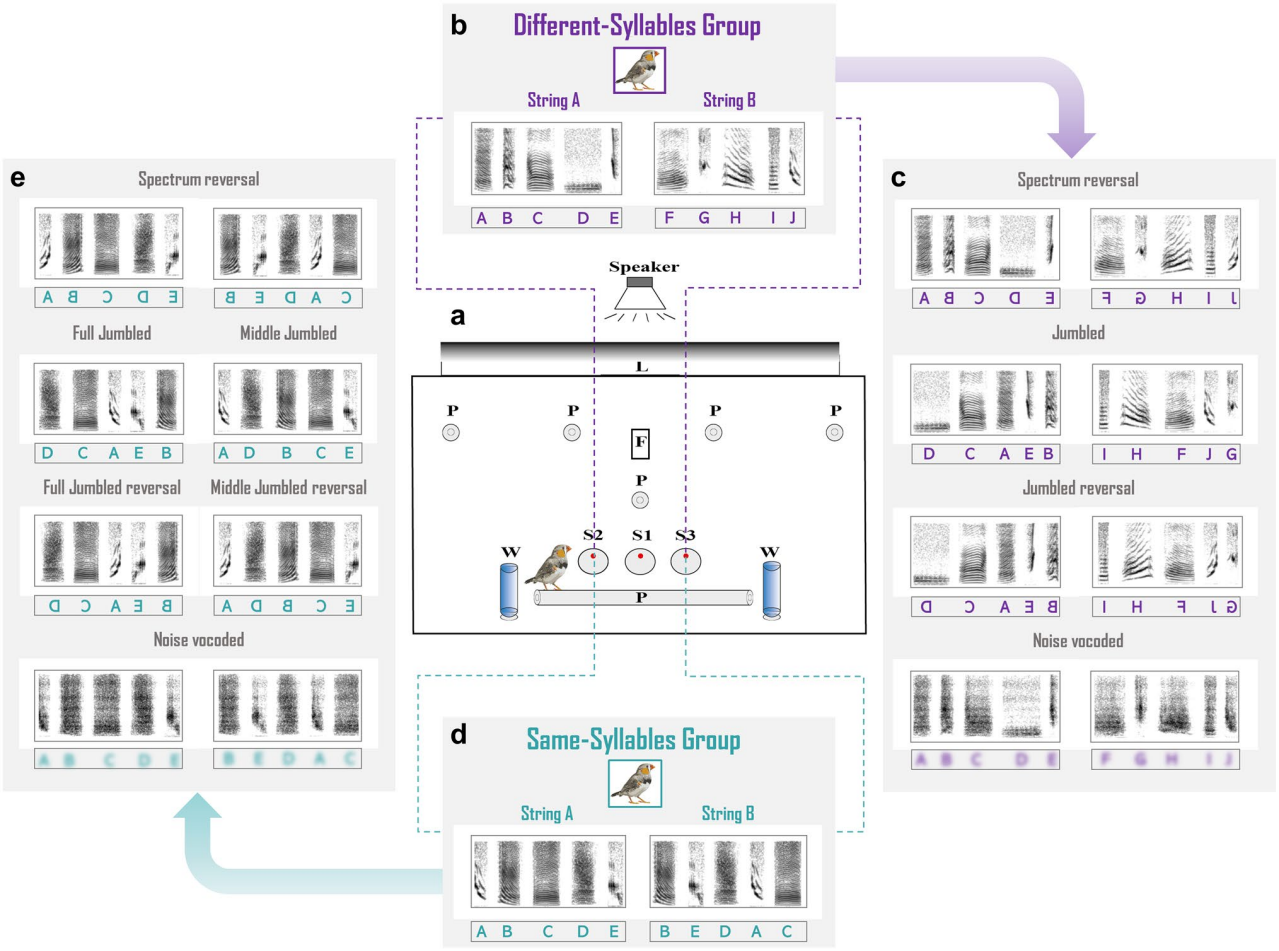
**a** Schematic view of the operant conditioning apparatus (Skinner box) used for the experiment. A speaker is suspended from the ceiling above the cage. Within the cage, there are several perches (P) for the bird to sit on, a food hatch (F) is located in the upper middle of the back panel, a lamp (L) is placed at the top of the cage. Two tubes of ad libitum water (W) are placed symmetrically on two sides of the cage, three response keys (S1, S2, S3) with signal LEDs are lined horizontally in the lower middle of the back panel. **b** An example of a pair of training strings for the Different-syllables group. The birds of the Different-syllables group were trained with stimuli consisting of different syllable types: for instance, String A was the sequence of syllables A B C D E, while String B was the sequence of syllables F G H I J. **c** Modified stimuli used in the testing phase for the Different-syllables group. The birds of the Different-syllables group were tested with 4 modified versions of each training stimulus after completion of the training—see text for a description of these manipulations. **d** A pair of training strings for the Same-syllables group. For birds of the Same-syllables group, training stimuli consisted of the same syllables, but arranged in different sequences: for instance, String A and String B consisted of the same five syllables A B C D E, but the sequences of these syllables were different between the two strings. **e** Modified stimuli in the testing phase for the Same-syllables group. These birds were also tested with 4 similarly modified versions of each training stimulus

The birds were trained and tested individually in an operant conditioning cage (Skinnerbox) (70 × 30 × 45 cm) using a Go-Left/Go-Right paradigm for training and testing. A cage contained 3 pecking keys (sensors) with a red LED light at the top/bottom of each sensor (Fig. 1a). Each operant cage was situated in a separate sound-attenuated chamber. The chamber was illuminated by a fluorescent lamp (Phillips Master TL-D 90 DeLuxe 18W/ 965, The Netherlands), which emitted a daylight spectrum following a 13.5-h/10.5-h light/dark schedule. Sound stimuli were played through a speaker (Vifa MG10SD09–08) 1 m above the Skinnerbox. The volume of the speaker was adjusted to ensure that the sound amplitude in the Skinnerbox was approximately 65 dB (measured by an SPL meter, RION NL 15, RION). Sensors (S1, S2, S3), lamp, food hatch and speaker were connected to the operant conditioning controller that also registered all sensor pecks.

### Stimuli

#### Training stimuli

Zebra finch syllables were selected from representative song recordings of adult males of the laboratory colony at Leiden University. The songs had not been heard before by the birds. Each string was composed of syllables belonging to different types, based on several distinctive acoustic features like the duration and spectral shape, mainly guided by the descriptions of syllable types in Lachlan et al. (2016). Each training string thus consisted of five song units, each of which belonged to one of in total 13 types of single-element syllables and 8 types of complex syllables. Each bird got different combinations of syllable types as training stimuli.

The five syllables within one string were normalized in root-mean-square (RMS) amplitude and separated by a 30-ms silent interval between each two syllables to form a natural song-syllable string. The training stimuli in this experiment were 24 stimulus pairs (12 pairs for each training group), each consisting of two different strings. For the Different-syllables group, each bird was presented with a stimulus pair of which the two strings consisted of different syllable types (Fig. 1b). For the Same-syllables group, each bird was presented with a stimulus pair of which the two strings consisted of a same set of syllables but arranged in a different sequence (Fig. 1d). To this end, we altered the syllable sequences of string A (indicated as “A–B–C–D–E”) into a different sequence “B–E–D–A–C” to construct the string B, which also avoids bigrams of syllables from string A.

When played, the strings were normalized such that the average intensity (RMS, calculated over the total duration of the stimulus) was the same for the two strings within a pair to avoid that amplitude differences affected the responses to the stimuli. The range of variation in volume recorded at the microphone was preserved. All stimuli were filtered to a bandwidth below 15 kHz. All training stimuli were cut, synthesized, and filtered using Praat (version 6.1.12). The amplitude of each stimulus was adjusted by using the “Normalize” function in Audacity (version 2.3.0).

#### Test stimuli

To test the impact of spectral and sequential information that the birds used to discriminate the training strings, they were tested with modified versions of the training strings (Fig. 1b, d). We used Praat to modify each original training string to produce a version in which either the spectral features or the sequence of syllables was changed. For each training group modified stimuli were changed in an identical way (some examples of the training and test stimuli are provided as supplementary material):

- SpectrumReversal: The spectrum of each syllable in the string was reversed, but the sequence of the syllables was identical to the order in the training version. We used the “reverse selection” option in Praat to reverse the spectrum of each syllable of a training string without changing the initial order.
- Jumbled: The sequence of the syllables in the training strings of both training groups were altered from “A–B–C–D–E” to “D–C–A–E–B”. For instance, if the syllable sequence of the string A in the Different-syllables group is “A–B–C–D–E”, then the order manipulated version becomes “D–C–A–E–B”, and the manipulated version of string B (the original sequence “F–G–H–I–J”) becomes “I–H–F–J–G”. Thus the “Jumbling” was applied to both string A and string B in the Different-syllables group (Fig. 1c). Likewise, this modification was applied in the Same-syllables group, by which the sequence-manipulated version of string A became “D–C–A–E–B”, and the sequence manipulation of string B became “A–D–B–C–E”. Note that this means that the manipulated string B now has the same first and fifth syllables as present in training string A (“A–B–C–D–E”), since training string A and string B consisted of the same syllables. Therefore, for the Same-syllables group, we distinguished in our analysis between the responses to “D–C–A–E–B”, which will be indicated as the “Full jumbled” test string and “A–D–B–C–E” which will be indicated as “Middle jumbled” test string, and we relate the responses to these test stimuli to the responses to training string A (Fig. 1e).
- Jumbled + SpectrumReversal: This manipulation was the combination of the above Jumbled alteration and SpectrumReversal. Both the spectrum of syllables and their sequence were changed (Fig. 1b, d).
- Vocoded: This modification maintains the spectral (and temporal) envelope of the syllables within the string, but averages the energy within specific frequency bands, thus removing any harmonic structure. To construct these stimuli, we used the Matt Winn's Praat vocoded script (http://www.mattwinn.com/praat/vocode_all_selected_v45.txt) to synthesize a vocoded morph of training strings. The script was set to divide cutoff frequency bandwidths equally for 15 bands contiguous with smooth transitions (1000 Hz bandwidth for one noise-vocoded band).

### Procedure

We used a Go-Left/Go-Right paradigm for training and testing (Fig. 1a). The training consisted of several phases.

#### Acclimation and pre-training

In the acclimation phase, the birds were moved to the Skinner boxes. The food hatch remained open, so food was freely accessible in a container behind the hatch. The LED lights on the pecking sensors were on. The goal of this phase was to acclimate the bird to the cage and to show it where to find food. The bird might also already learn to peck sensors spontaneously. If in this stage the central sensor, S1, was stimulated by pecking, it would play sound string A or sound string B with a 50% chance on each. The side sensor S2 produced one of the two training strings, and the other side sensor S3 produced the other string. The LEDs of all three sensors were illuminated to attract the attention from the bird. After a few hours to one night of acclimation, the pre-training phase started by closing the food hatch. In this phase, the food hatch was closed, and the bird had to learn to peck at each sensor, and that pecking the sensors resulted in access to the food. The bird might also already learn at this stage which song was related to S2 or S3. Once the bird started to peck all the sensors regularly for a day, the discrimination training phase began.

#### Discrimination training

In this phase, the bird had to learn to peck the sensor in the middle to elicit the playback sound, and then to peck S2 or S3, depending on the playback sound. If the bird pecked the sensor that was linked to the stimulus being played, this was rewarded with 12 s access to food. If the wrong sensor was pecked the light was off for 1 s. Before any sensor was pecked, only the S1 LED was on. If the bird did not respond within 15 s, a trial would end automatically without food reward or light-off penalty. The duration of this phase varied from bird to bird (range 5–32 days). The proportion of correct responses (see ‘Analysis’ for calculation of the ‘Correct rate’) was calculated on a daily basis as the individual's discrimination rate among the training stimuli.

#### Transition phase

When a bird learned to associate the two training sounds with the corresponding sensors and had reached a Correct rate for the training stimuli greater than 0.75 for three consecutive days, it was assumed that the bird was able to discriminate the trained song motifs and the training was switched to a transition phase, in which the reinforcement by food reward or darkness was reduced to occur randomly on 80% (instead of 100%) of trials. On the remaining 20% of trials, the responses were not reinforced, and the trial ended after 15 s. If the bird kept the same level of discrimination for 2 days, the test phase began.

#### Probe testing phase

In this phase, 20% of the pecks on S1 resulted in presenting one of ten test stimuli. These ten test stimuli were never reinforced and were randomly interspersed between training stimuli. Eight of these were modified versions of the training stimuli (four modified versions of stimulus A and four of stimulus B). The other two were non-reinforced training stimuli. The remaining 80% were training stimuli with reinforcement. Testing continued until each test stimulus had been presented 40 times to a bird. After reaching this, the bird was transferred back to its aviary. The order of stimulus presentation was random across subjects.

### Analysis

For the speed of discrimination learning, we used the total number of trials up to and including the day on which the learning criterion had been reached. A two-tailed unpaired *t* test (using the *t* test function in GraphPad Prism 9.1.1) was used to detect differences between the two training groups.

The reactions to the different test stimuli can be separated into three categories: a ‘correct response’ (i.e., the bird identifies the modified version of training stimulus A as A and the modified version of training stimulus B as a B), an ‘incorrect response’ (responding with pecking the sensor for B if the stimulus was a modification of sound A and vice versa), and a ‘no response’ (not pecking a key). For the statistical analyses, we examined the proportion of ‘correct responses’ out of ‘correct + incorrect responses’ (Correct rate = Count_Correct/(Count_Correct + Count_Incorrect)), as well as the 'response rate', i.e. the proportion of ‘correct + incorrect responses’ to modifications of sound A plus those to modification of sound B, out of the 40 presentations of each test stimulus (Response rate = (Count_Correct + Count_Incorrect)/(Count_Correct + Count_Incorrect + Count_NoResp)). In addition, we examined whether the individual test stimuli were discriminated above chance.

We used generalized linear mixed-effects models (GLMMs) to examine the discrimination of various test sounds by the birds. All model analyses were conducted in Rstudio (R Core Team 2016). We calculated the ‘Correct rate’ and the ‘Response rate’ based on the counts of ‘correct response’, ‘incorrect response’, and ‘no response’, combining the response counts to (variants of) Training strings A and B, using the function cbind, R package mice; Van Buuren and Groothuis-Oudshoorn 2011, and used these two rates as response variables in GLMMs in R (using the function glmer, R package lme4; Bates et al. 2015). We used ‘Training_Group’ (same or different syllables), ‘Test_Treatment’, and the interaction between these two as covariates in the full model with ‘Bird_ID’, ‘Age’, ‘Number_of_Training_Trials’ as the random factors and a binomial error structure of the ‘Correct rate’ and the ‘Response rate’. The best model was chosen based on corrected Akaike information criterion (AICc) provided by dredge model selection (using the function Dredge, R package MuMIn; Bartoń 2020). The model with the smallest value of AICc was considered to be the best model by default, but if ‘Training_Group’ was not part of the best model, we kept it in the final model anyway because this was a variable of our interest. To determine the effect and significance of the covariates, we ran the final models and, if applicable, used post hoc Tukey's HSD tests to make pairwise comparisons of the test treatments (using the emmeans function, R package lsmeans; Lenth 2016), with false discovery rate (FDR) correction of *p* values (Benjamini and Hochberg 1995) for multiple comparisons.

In the above model, the counts of the responses to (modifications of) both string A and string B were combined in all tests. This included the two test treatments ‘Jumbled’ and ‘JumbledReversal’ for both string A and B in the Same-syllables group. As outlined above, however, the jumbling of the syllables resulted in making the jumbled version of string B partly similar to training string A, and we therefore used string A as reference in this case. Because jumbling the strings for the Same-syllables group thus resulted in half of the jumbled strings being fully jumbled and the other half being middle jumbled, we also did a separate analysis for the data set of two Jumbled versions (MiddleJumbled/FullJumbled) in the Same-syllables training group. In this analysis we compared the responses to training string A with those to the FullJumbled version of string A and those to the MiddleJumbled version in which the 1st and 5th syllables of the test string are the same as those of the training string A. In this analysis, ‘Test_Treatment’ was used as a fixed effect in the full model to gain insight into a possible comparison among three different stimuli versions (Training/MiddleJumbled/FullJumbled). The ‘Bird_ID’, ‘Age’, and ‘Number_of_Training_Trials’ were included as the random factors. Here we also used a model with binomial error structure of the Correct and the Response rates.

To examine whether the birds responded above chance (50%) to each of the testing stimuli, we applied a log(correct/incorrect) as the response variables against a log (odds-ratio) = 0 in a GLM. If correct/incorrect = 1, then the probability of observing a correct response is as large as the probability of observing an incorrect response, with both probabilities being 0.5, in which case log (odds ratio) = log (1) = 0. Therefore, comparing the outcomes of the binomial GLM to 0 is comparing the results to the 50% chance for a correct response.

### Ethics statement

All animal housing, care, and use was approved by the national *Centrale Commissie voor Dierproeven* (CCD) of the Netherlands and the Leiden University Animal Welfare Body (AVD number 1060020197507). None of 24 birds had any experience with this experimental setup or the stimuli preceding the experiment. Each experimental bird underwent a physical examination before being transferred to the Skinnerboxes. During the experiment, the health and welfare of these birds was monitored daily. The food intake of the birds was monitored daily, and additional food was given when there were signs of a low food intake.

## Results

### Learning speed

**Fig. 2:**
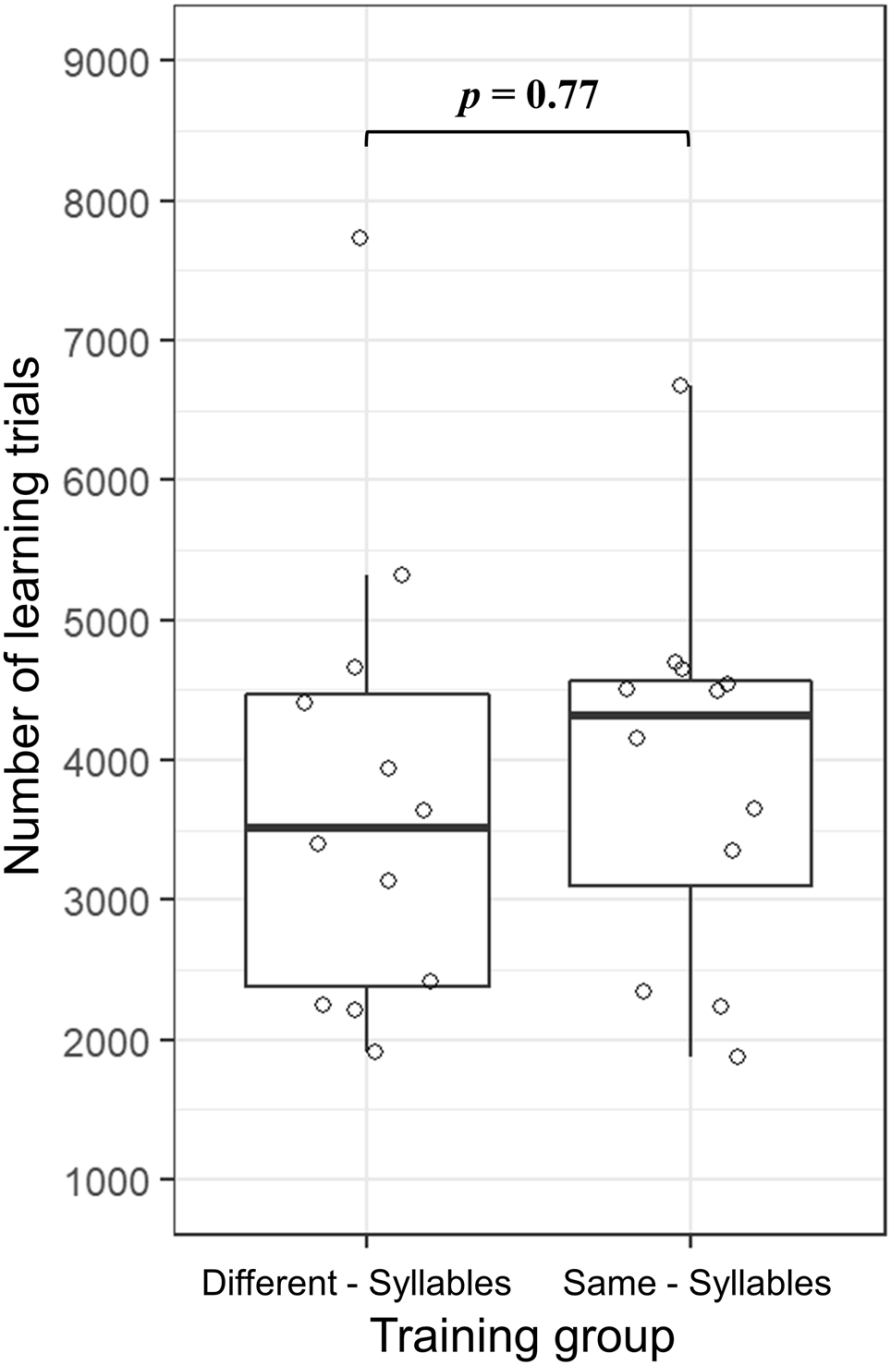
Number of learning trials needed to reach the learning criterion. Individual zebra finch results are shown with open circles. There is no significant difference between the Different-syllables group and the Same-syllables group in learning speed. Box plots show median, first and third quartile, and whiskers the 1.5 interquartile range

The discrimination training lasted until the birds reached the learning criterion of over 75% correct responses to both sound A and sound B for three successive days. All twenty-four birds finished the training and reached the learning criterion in, on average, 3842 (SD = 1442, *N* = 24) trials. No significant difference (*p* = 0.7733, *t* = 0.2916, *df* = 22; Fig. 2) was found between the Different-syllables group (M = 3753, SD = 1579) and the Same-syllables group (M = 3932, SD = 1283). It suggests that birds from two training groups learned approximately equally fast.

### Do training groups differ in responses to test stimuli?

**Fig. 3:**
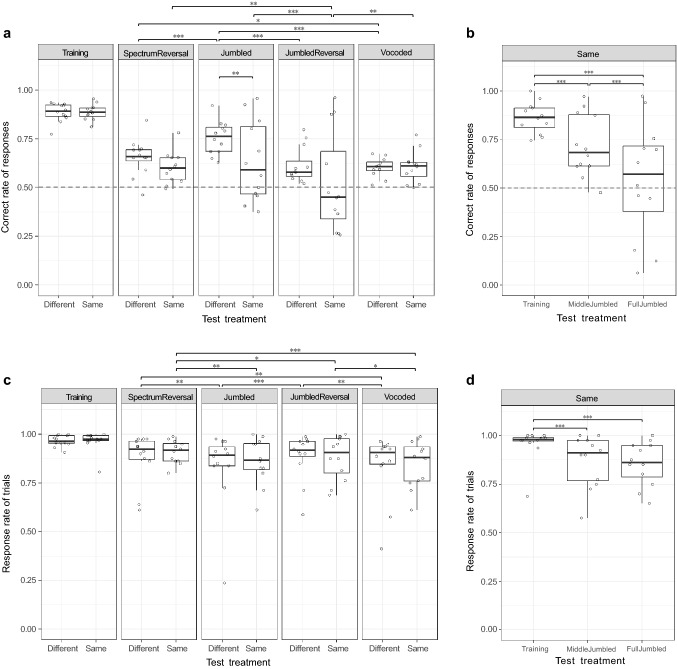
Correct rate of responses and Response rate to test stimuli. **a** The Correct rate of responses to the training and test stimuli for the two training groups; **b** the Correct rate of responses to the training stimulus and the two Jumbled versions for the ‘Same-syllables’ training group; **c** the Response rates to the training and test stimuli for the two training groups; **d** the Response rates to the training stimulus and the two Jumbled versions for the ‘Same-syllables’ training group. All test stimuli got significantly lower Correct rates and significantly lower Response rates than the training stimuli. Significant differences between the responses to the various test stimuli and between the training groups are indicated: *** refers to a significant difference of *p* ≤ 0.001, ** refers to a significant difference of 0.001 < *p* ≤ 0.01, and * refers to a significant difference of 0.01 < *p* ≤ 0.05, for non-indicated comparisons *p* value is > 0.05. Box plots show median, first and third quartile, and whiskers the 1.5 interquartile range. The dashed line represents chance level, which was 50% for both tasks

We compared the Correct rates and Response rates to the training and various test stimuli in both experimental groups (Fig. 3). For the Correct rate, the best model (model 1) was chosen based on AICc (Table 1). For the Response rate, we chose the model 3 with the same factors as model 1 for the Correct rate. It was not the most recommended model by the dredge model selection, but it contained the variables of our interest and was also close to the most recommend model (AICc = 723.1, delta = 7.41, Table 1).

**Table 1:** Summary of the GLMs selection for (a) the proportion of correct responses if birds respond to one of two sounds; and (b) the proportion of trials that birds respond with pecking A or B

Model	*df*	logLik	AICc	Δi	wi
(a) Correct rate of responses (sound A + B combined)					
1* Training_Group + Test_Treatment + Test_Treatment: Training_Group + (1|Bird_ID) + (1|Age) + (1|Number_of_Training_Trials)	13	− 481.009	991.5	0.00	0.964
2 Training_Group + Test_Treatment + (1|Bird_ID) + (1|Age) + (1|Number_of_training_trials)	9	− 489.805	999.2	7.79	0.020
3 Test_Treatment + (1|Bird_ID) + (1|Age) + (1|Number_of_Training_Trials)	8	− 491.141	999.6	8.13	0.017
4 Training_Group + (1|Bird_ID) + (1|Age) + (1|Number_of_Training_Trials)	5	− 789.401	1589.3	597.88	0.000
null (1|Bird_ID) + (1|Age) + (1|Number_of_Training_Trials)	4	− 790.736	1589.8	598.37	0.000
(b) Response rate of trials (sound A + B combined)					
1 Test_Treatment + (1|Bird_ID) + (1|Age) + (1|Number_of_Training_Trials)	8	− 349.172	715.6	0.00	0.748
2 Training_Group + Test_Treatment + (1|Bird_ID) + (1|Age) + (1|Number_of_Training_Trials)	9	− 349.165	718.0	2.33	0.234
3* Training_Group + Test_Treatment + Test_Treatment: Training_Group + (1|Bird_ID) + (1|Age) + (1|Number_of_Training_Trials)	13	− 346.811	723.1	7.41	0.018
4 Training_Group + (1|Bird_ID) + (1|Age) + (1|Number_of_Training_Trials)	5	− 469.717	950.0	234.32	0.000
null (1|Bird_ID) + (1|Age) + (1|Number_of_Training_Trials)	4	− 469.723	947.8	232.15	0.000

Best four models of the model selection (ranked by AICc and logLik) and the null models. The Akaike weight (wi) indicates the probability of a better model in the model candidates set, and Delta AICc (Δi) was used to show the difference in AICc score between the best model and the model being compared. A * indicates the model we choose. Only information related to both sound A and sound B are shown here; the information about the two Jumbled versions in the ‘Same-syllables’ training group is not displayed in this table

The only significant difference between the two training groups concerns the Correct rate for the Jumbled version (Different – Same = 0.534 ± 0.173, *p* = 0.01, Table 2). There were no significant differences in the Correct rate for any of the other test stimuli between the two training groups (Fig. 3a). Note that the variation in Correct rate for the Jumbled test stimuli in the Same-syllables group is much larger than that for other test stimuli, which is caused by combining the responses to both the ‘Middle Jumbled’ and ‘Full Jumbled’ test stimuli (see below for the analysis separating among these stimuli). There were no significant differences in Response rates for any of the stimuli between two training groups (Fig. 3c).

**Table 2:** Post hoc test results of binomial GLMMs for the interaction of Test and Training_Group

Stimuli	Training_Group	Estimate	SE	*z* ratio	*p* value
(a) Correct rate of responses (sound A + B in two training groups)					
Training	Different—Same	− 0.007	0.198	− 0.036	0.9715
SpectrumReversal	Different—Same	0.255	0.167	1.526	0.2117
**Jumbled**	**Different—Same**	**0.534**	**0.173**	**3.094**	**0.0100**
JumbledReversal	Different—Same	0.350	0.166	2.108	0.0878
Vocoded	Different—Same	− 0.020	0.168	− 0.119	0.9715
**Training—SpectrumReversal**	**Different**	**1.423**	**0.127**	**11.200**	** < 0.0001**
**Training—Jumbled**	**Different**	**0.958**	**0.133**	**7.217**	** < 0.0001**
**Training—JumbledReversal**	**Different**	**1.615**	**0.126**	**12.823**	** < 0.0001**
**Training—Vocoded**	**Different**	**1.673**	**0.127**	**13.188**	** < 0.0001**
**SpectrumReversal—Jumbled**	**Different**	− **0.465**	**0.110**	− **4.228**	** < 0.0001**
SpectrumReversal—JumbledReversal	Different	0.192	0.102	1.884	0.0662
**SpectrumReversal—Vocoded**	**Different**	**0.251**	**0.103**	**2.434**	**0.0186**
**Jumbled—JumbledReversal**	**Different**	**0.657**	**0.109**	**6.045**	** < 0.0001**
**Jumbled—Vocoded**	**Different**	**0.716**	**0.110**	**6.528**	** < 0.0001**
JumbledReversal—Vocoded	Different	0.059	0.102	0.578	0.5631
**Training—SpectrumReversal**	**Same**	**1.684**	**0.125**	**13.434**	** < 0.0001**
**Training—Jumbled**	**Same**	**1.499**	**0.127**	**11.781**	** < 0.0001**
**Training—JumbledReversal**	**Same**	**1.971**	**0.126**	**15.711**	** < 0.0001**
**Training—Vocoded**	**Same**	**1.660**	**0.127**	**13.076**	** < 0.0001**
SpectrumReversal—Jumbled	Same	− 0.185	0.102	− 1.821	0.0857
**SpectrumReversal—JumbledReversal**	**Same**	**0.287**	**0.099**	**2.889**	**0.0055**
SpectrumReversal—Vocoded	Same	− 0.024	0.101	− 0.238	0.8117
**Jumbled—JumbledReversal**	**Same**	**0.472**	**0.102**	**4.642**	** < 0.0001**
Jumbled—Vocoded	Same	0.161	0.104	1.555	0.1334
**JumbledReversal—Vocoded**	**Same**	− **0.311**	**0.101**	− **3.070**	**0.0036**
(b) Response rate of trials (sound A + B in two training groups)					
Training	Different—Same	0.118	0.467	0.252	0.9724
SpectrumReversal	Different—Same	− 0.221	0.420	− 0.525	0.9724
Jumbled	Different—Same	− 0.185	0.412	− 0.448	0.9724
JumbledReversal	Different—Same	0.156	0.418	0.374	0.9724
Vocoded	Different—Same	0.014	0.411	0.035	0.9724
**Training—SpectrumReversal**	**Different**	**1.452**	**0.209**	**6.937**	** < 0.0001**
**Training—Jumbled**	**Different**	**1.896**	**0.204**	**9.291**	** < 0.0001**
**Training—JumbledReversal**	**Different**	**1.393**	**0.210**	**6.630**	** < 0.0001**
**Training—Vocoded**	**Different**	**1.859**	**0.204**	**9.097**	** < 0.0001**
**SpectrumReversal—Jumbled**	**Different**	**0.444**	**0.143**	**3.108**	**0.0027**
SpectrumReversal—JumbledReversal	Different	− 0.059	0.152	− 0.384	0.7790
**SpectrumReversal—Vocoded**	**Different**	**0.407**	**0.143**	**2.841**	**0.0056**
**Jumbled—JumbledReversal**	**Different**	− **0.502**	**0.144**	− **3.483**	**0.0010**
Jumbled—Vocoded	Different	− 0.037	0.134	− 0.272	0.7856
**JumbledReversal—Vocoded**	**Different**	**0.466**	**0.145**	**3.217**	**0.0022**
**Training—SpectrumReversal**	**Same**	**1.114**	**0.214**	**5.197**	** < 0.0001**
**Training—Jumbled**	**Same**	**1.594**	**0.206**	**7.746**	** < 0.0001**
**Training—JumbledReversal**	**Same**	**1.432**	**0.208**	**6.879**	** < 0.0001**
**Training—Vocoded**	**Same**	**1.756**	**0.204**	**8.621**	** < 0.0001**
**SpectrumReversal—Jumbled**	**Same**	**0.480**	**0.150**	**3.191**	**0.0024**
**SpectrumReversal—JumbledReversal**	**Same**	**0.319**	**0.154**	**2.071**	**0.0480**
**SpectrumReversal—Vocoded**	**Same**	**0.642**	**0.148**	**4.353**	** < 0.0001**
Jumbled—JumbledReversal	Same	− 0.1616	0.141	− 1.142	0.2534
Jumbled—Vocoded	Same	0.1623	0.135	1.206	0.2531
**JumbledReversal—Vocoded**	**Same**	**0.3239**	**0.138**	**2.341**	**0.0275**

Response variables in GLMMs: (a) the proportion of correct responses if birds respond to one of two sounds; and (b) the proportion of trials that birds respond with pecking A or B. Only information related to both sound A and sound B is shown here, the information about the two Jumbled versions in the ‘Same-syllables’ training group is not displayed in this table. Bold indicates significance

### Do different test stimuli give rise to different responses?

The highest Correct and Response rates are present for the non-rewarded training stimuli. Thus, in both training groups all modifications affected the birds' responses (see Table 2). For the comparisons of responses to different test stimuli within each training group, Post hoc Tukey's HSD tests (Table 2) showed that the birds responded with a higher Correct rate and a higher Response rate to the training stimuli compared to all four testing stimuli in both training groups (Fig. 3a, c). The tests also showed that the birds of the Different-syllables training group responded with a significantly higher Correct rate to the Jumbled stimuli than to the JumbledReversal, the Vocoded stimuli and the SpectrumReversal stimuli (both *p* < 0.0001), and with a significantly higher Correct rate to the SpectrumReversal stimuli than to the Vocoded stimuli (*p* < 0.05), while the birds of the Same-syllables training group responded with a significantly lower Correct rate to the JumbledReversal stimuli than to the Jumbled stimuli (*p* < 0.0001), the Vocoded stimuli and the SpectrumReversal stimuli (both *p* < 0.01).

The birds of the Different-syllables training group had lower Response rate to the Jumbled stimuli and the Vocoded stimuli than to the JumbledReversal (*p* < 0.01), and had a significantly higher Response rate to the SpectrumReversal stimuli than to the Jumbled stimuli and the Vocoded stimuli (both *p* < 0.01), while the birds of the Same-syllables training group had significantly lower Response rate to the JumbledReversal (*p* < 0.05), the Vocoded (*p* < 0.0001) and the Jumbled stimuli (*p* < 0.01) than to the SpectrumReversal stimuli, and had a significantly higher Response rate to the JumbledReversal than to the Vocoded stimuli (*p* < 0.05).

To investigate the impact on discrimination of the two Jumbled versions in the Same-syllables training group, we split the data for the responses to the Jumbled version into responses to the MiddleJumbled version and FullJumbled version, comparing them with the responses given to training sound A. This showed that the birds responded with a higher Correct rate to Training sound A than to the MiddleJumbled test sound and with a higher Correct rate to the MiddleJumbled than to the FullJumbled test sound (Training—MiddleJumbled = 0.9071 ± 0.1812, MiddleJumbled—FullJumbled = 0.9094 ± 0.1603, both *p* < 0.001) (Fig. 3b). There was no significant difference in the Response rate between these two Jumbled versions (MiddleJumbled—FullJumbled = 0.1404 ± 0.2004, *p* = 0.76), but both rates were lower than the Response rate to Training sound A (Training—MiddleJumbled = 1.3877 ± 0.2809, Training—FullJumbled = 1.5281 ± 0.2783, both *p* < 0.001) (Fig. 3d). These results (see Table S1 in the supplementary appendix) show that the birds of the ‘Same-syllables’ training group pay attention to the beginning and end, as well as to the middle syllables of the strings.

### Are modified stimuli still discriminated?

**Fig. 4:**
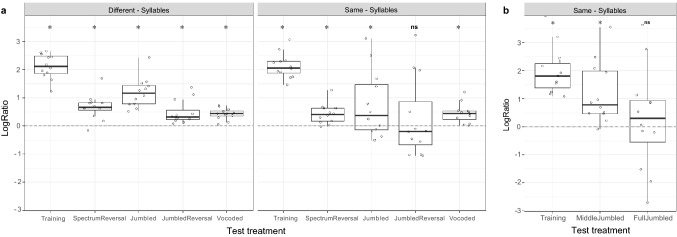
Visualization of logRatios = log (correct/incorrect). **a** For the Different-syllable group (left), all logRatios are statistically different from zero; for the Same-syllable group (right), the Test treatment JumbledReversal is not significantly different from 0; **b** Results for the Jumbled test sounds of the Same-syllable group, split into Middle and FullJumbled. For MiddleJumbled, there is a small overlap with zero; for Jumbled, it is statistically not different from 0. A * indicates that the logRatio of a Test treatment is significantly different from 0, ‘ns’ indicates that the logRatio of a test treatment overlaps with 0. Box plots show median, first and third quartile, and whiskers the 1.5 interquartile range. Horizontal dashed lines show the discrimination boundaries in which the proportion of correct responses is equal to the proportion of incorrect responses. The calculation of logRatios was based on the counts of ‘correct response’ and ‘incorrect response’ from the same data set that was also used for Fig. 3

The above analyses concentrated on differences in the Correct rates between the groups and among the test stimuli. They do not test whether a low Correct rate also indicates that birds no longer discriminate between the modified version of training sound A and that of the similarly modified version of training sound B. If the birds are still capable of linking the modified stimuli to the respective training stimuli, the proportion of correct responses to the test stimuli should be higher than the proportion of incorrect responses. Table 3 and Fig. 4a show that for the Different-syllables group, all treatment combinations are significantly different from 0 in favor of a correct response. For the Same-syllables group, all treatments were also statistically different from 0 in favor of correct response, except the Test treatment JumbledReversal, which showed no significant difference from 0 (Fig. 4a).

**Table 3:** Lower and upper 95% confidence limits (CL) of the confidence interval

Training group	Stimuli	Estimate	SE	CL (95%)	
				Lower	Upper
LogRatio ~ Training_Group + Test_Treatment + Test_Treatment: Training_Group + (1|Bird_ID) + (1|Age) + (1|Number_of_Training_Trials), data = sound A + sound B, *n* = 24					
**Different syllables**	**Training**	**2.090**	**0.142**	**1.812**	**2.368**
**Different syllables**	**SpectrumReversal**	**0.667**	**0.121**	**0.430**	**0.904**
**Different syllables**	**Jumbled**	**1.132**	**0.127**	**0.883**	**1.381**
**Different syllables**	**JumbledReversal**	**0.475**	**0.120**	**0.241**	**0.710**
**Different syllables**	**Vocoded**	**0.417**	**0.121**	**0.180**	**0.653**
**Same syllables**	**Training**	**2.097**	**0.142**	**1.819**	**2.375**
**Same syllables**	**SpectrumReversal**	**0.412**	**0.119**	**0.179**	**0.646**
**Same syllables**	**Jumbled**	**0.597**	**0.121**	**0.360**	**0.835**
Same syllables	JumbledReversal	0.125	0.119	− 0.108	0.359
**Same syllables**	**Vocoded**	**0.436**	**0.121**	**0.200**	**0.673**
LogRatio ~ Test_Treatment + (1|Bird_ID) + (1|Age) + (1|Number_of_Training_Trials), data = sound A, *n* = 12					
**Same syllables**	**Training**	**2.073**	**0.309**	**1.468**	**2.678**
**Same syllables**	**MiddleJumbled**	**1.166**	**0.298**	**0.581**	**1.751**
Same syllables	FullJumbled	0.257	0.293	− 0.319	0.832

If zero is part of the confidence interval, the treatment combination training group and stimuli are not significantly different from 0. If both confidence levels are positive, then there is a bias toward correct responses. If they are both negative, then they are more biased toward incorrect responses. Bold indicates significance

For the data set of two Jumbled versions in Same-syllables group, MiddleJumbled is statistically different from 0 in favor of correct response, but FullJumbled is not significant different from 0 (Table 3), which is in line with the visualization (Fig. 4b).

## Discussion

Our results show that zebra finches are capable of using both spectral features and sequential information to discriminate strings consisting of conspecific song syllables. Confirming results obtained in earlier studies on zebra finches, our study also demonstrates that zebra finches will give higher priority to using spectral features than to syllable sequence in discrimination when the syllables differ in phonology. When strings are composed of the same set of syllables, zebra finches learn about the syllable sequence in addition to the syllable phonology.

### No effect of stimulus composition on learning speed

Various studies (Braaten et al. 2006; Lawson et al. 2018; Geberzahn and Derégnaucourt 2020) demonstrated that when zebra finches learned to discriminate between two songs, they were very sensitive to changes in the spectral domain (syllable reversals) and hardly sensitive to sequential information (sequence reversals), similar to what we observed in our ‘Different-syllables’ training group. These studies indicated that the zebra finches ignored sequence cues in discrimination learning or that sequences were more difficult to learn than spectral features and might require more time. In line with this, some studies (Lawson et al. 2018; Braaten et al. 2006) indicated that if zebra finches used syllable sequences to distinguish songs, this occurred with songs to which the birds had been exposed more extensively. That learning to discriminate sequences consisting of the same sets of syllables might be more difficult than sequences consisting of different syllables was also suggested by a meta-analysis using data from 14 different acoustic Go/No-go experiments with zebra finches (Kriengwatana et al. 2016), which indicated that stimuli (either zebra finch vocalizations or human speech syllables) differing in phonetic characteristics were learned faster than those differing in sequence only. However, in our experiment, which allowed a direct comparison of learning speeds of comparable stimuli in identical conditions, the learning speed of the training group relying only on sequence cues is not significantly lower than that of the group trained on stimuli with different syllables. This suggests that the Same-syllables group learned about the syllable sequence in parallel with learning about the syllable phonology, without requiring more extensive exposure or training.

### Cognitive flexibility in processing syllable phonology and sequence

The comparison of the correct responses to the different test stimuli showed that both training groups were similarly strongly affected by changes of the spectro-temporal features of the syllables, thus noticing such changes equally well. It demonstrates that the Same-syllables group, which can only learn a sequence of syllables when they also learn the spectro-temporal features of these syllables, gives the same weight to the spectro-temporal features as the Different-syllables group does. The difference between the two training groups concerns their responses to the jumbled test sounds. Although the jumbled test stimuli received fewer correct responses and had a lower Response rate than the training stimuli in both groups, jumbling affected the Same-syllables group much more strongly than the Different-syllables group. For the Same-syllables group, the impact of jumbling is similar to that of spectral changes. Jumbling had a lesser impact than spectral modifications in the Different-syllables group, confirming that this group mainly (although not exclusively) relied on spectral features of the syllables to distinguish the training strings. Hence, the importance of syllable sequence increased when knowledge of the sequence is needed to correctly identify different strings. This finding indicates the presence of ‘cognitive flexibility’ in processing string information, in which sequence learning can be added to learning of spectro-temporal features of syllables when needed to distinguish strings.

No differences were observed between the responses of both groups to reversal of the syllables and vocoding them. Reversal of syllables reverses the within-syllable spectral and amplitude pattern (i.e., any frequency changes or increasing or decreasing amplitude over an element), while vocoding maintains these patterns, but removes pitch information. Apparently, all these dimensions are taken into account for identification of syllables. Nevertheless, both groups were capable of still discriminating reversed and vocoded versions of the training stimuli, indicating that the test stimuli still maintained sufficient gross spectral differences among the syllables of a string to allow for string identification.

That full jumbling strongly affected the Same-syllables group and resulted in absence of discrimination is no surprise, as full jumbling removed all information that might relate to the original syllable sequences. However, what is of interest is that middle jumbled also got fewer correct responses than the training stimuli, indicating that the birds were not just relying on the first and last syllables of the syllable sequence (which was suggested by studies on zebra finches (Fishbein et al. 2019) and Bengalese finches (*Lonchura striata *var.* domestica*) (Mizuhara and Okanoya 2020)) but also to the sequence of the middle syllables.

### Vocal production learning and discrimination learning

Altogether, the results indicate that sequence learning can be ‘added to’ learning about spectro-temporal features of syllables if these features alone are insufficient to distinguish two syllable strings. It indicates the presence of sequence learning as a separate, but nevertheless strongly connected or partially overlapping learning process, similar to what has been observed in several studies of song production learning (Liu et al. 2004; Braaten et al. 2006; Lipkind et al. 2013, 2017). This does not imply that song production learning and song discrimination learning rely on the same mechanisms. Song production learning occurs in male zebra finches only and only during a sensitive phase early in life, while discrimination learning can occur in both sexes and when adult. Also, vocal discrimination learning has been observed in vocal non-learning species, such as dove species (Beckers and ten Cate 2001; Beckers et al. 2003), which give attention to both spectral and temporal structure of sound strings. Hence, vocal production learning and later-occurring vocal discrimination or recognition learning are likely to rely at least partly on different mechanisms.

In conclusion, our study demonstrates that although zebra finches have a bias to attend to spectral features when recognizing or discriminating strings of syllables, they can also attend to the sequence when needed. Our study did not test whether the relative importance of syllable sequence might vary if the syllable similarity between strings also varies, e.g., when not all but only part of the syllables in a string are different, or when different strings contain different exemplars of the same syllable types. It is likely that such string modifications may affect the relative weight of spectro-temporal and sequence parameters in song discrimination. Such flexibility may explain why some studies on the cues that zebra finches use to distinguish songs demonstrated absence of any impact of changes in syllable sequences on discriminating strings (Lawson et al. 2018; Geberzahn and Derégnaucourt 2020; Mol et al. 2021), while other studies (van Heijningen et al. 2009; Chen et al. 2016; Spierings and ten Cate 2016) showed clear sequence learning. It shows that the use of particular cues within a specific experiment should not be taken as an inability to use other cues when such cues might be useful or needed to correctly identify different strings, although the importance of the ability to also learn about syllable sequences under natural conditions remains to be elucidated. A similar flexibility, in this case for using different spectral cues, was observed by Burgering et al. (2018, 2019), showing that depending on the differences among training sounds, zebra finches used either pitch or spectral envelope to distinguish the training sounds. To what extent such flexibility is also present for other song features awaits further exploration. It is likely that zebra finches are not the only species that demonstrates such cognitive flexibility, although this remains to be tested. The benefit of such flexibility is that it may allow birds to adjust their perceptual tuning to those acoustic dimensions that are most relevant to distinguish songs of different individuals or other biologically relevant sounds.

## Supplementary Information

Below is the link to the electronic supplementary material.

Supplementary file1 (ZIP 688 KB)

Supplementary file2 (ZIP 663 KB)

Supplementary file3 (DOCX 18 KB)

## Data Availability

Samples of the audio stimuli are provided as electronic supplementary material. The datasets generated during and/or analyzed during the current study are available from the corresponding author on reasonable request.

## References

[CR1] Bartoń K (2020) MuMIn: multi-model inference. R package version 1.43.17. https://CRAN.R-project.org/package=MuMIn

[CR2] Bates D, Maechler M, Bolker B, Walker S (2015). Fitting linear mixed-effects models using lme4. J Stat Softw.

[CR3] Beckers GJL, ten Cate C (2001). Perceptual relevance of species-specific differences in acoustic signal structure in Streptopelia doves. Anim Behav.

[CR4] Beckers GJL, Goossens BMA, ten Cate C (2003). Perceptual salience of acoustic differences between conspecific and allospecific vocalizations in African collared-doves. Anim Behav.

[CR5] Benjamini Y, Hochberg Y (1995). Controlling the false discovery rate: a practical and powerful approach to multiple testing. J R Stat Soc Ser B.

[CR6] Braaten RF, Petzoldt M, Colbath A (2006). Song perception during the sensitive period of song learning in zebra finches (*Taeniopygia guttata*). J Comp Psychol.

[CR7] Burgering MA, ten Cate C, Vroomen J (2018). Mechanisms underlying speech sound discrimination and categorization in humans and zebra finches. Anim Cogn.

[CR8] Burgering MA, Vroomen J, ten Cate C (2019). Zebra finches (*Taeniopygia*
*guttata*) can categorize vowel-like sounds both on the fundamental frequency ("Pitch") and spectral envelope. J Comp Psychol.

[CR9] Cazala A, Giret N, Edeline JM, Del Negro C (2019). Neuronal encoding in a high-level auditory area: from sequence of elements to grammatical structure. J Neurosci.

[CR10] Chen J, ten Cate C (2015). Zebra finches can use positional and transitional cues to distinguish vocal element strings. Behav Processes.

[CR11] Chen J, ten Cate C (2017). Bridging the gap: Learning of acoustic nonadjacent dependencies by a songbird. J Exp Psychol Anim Learn Cogn.

[CR12] Chen J, Jansen N, ten Cate C (2016). Zebra finches are able to learn affixation-like patterns. Anim Cogn.

[CR14] Dooling RJ, Prior NH (2017). Do we hear what birds hear in birdsong?. Anim Behav.

[CR15] Eales LA (1985). Song learning in zebra finches: Some effects of song model availability on what is learnt and when. Anim Behav.

[CR16] Eens M (1997). Understanding the complex song of the European starling: an integrated ethological approach. Adv Study Anim Behav.

[CR17] Fishbein AR, Idsardi WJ, Ball GF, Dooling RJ (2019). Sound sequences in birdsong: how much do birds really care?. Philos Trans R Soc Lond B Biol Sci.

[CR18] Fishbein AR, Prior NH, Brown JA, Ball GF, Dooling RJ (2021). Discrimination of natural acoustic variation in vocal signals. Sci Rep.

[CR19] Geberzahn N, Derégnaucourt S (2020). Individual vocal recognition in zebra finches relies on song syllable structure rather than song syllable order. J Exp Biol.

[CR20] Gil D, Slater PJB (2000). Song organisation and singing patterns of the willow warbler, *Phylloscopus*
*trochilus*. Behaviour.

[CR21] James LS, Sakata JT (2017). Learning biases underlie "universals" in avian vocal sequencing. Curr Biol.

[CR22] Knowles JM, Doupe AJ, Brainard MS (2018). Zebra finches are sensitive to combinations of temporally distributed features in a model of word recognition. J Acoust Soc Am.

[CR23] Kriengwatana B, Spierings MJ, ten Cate C (2016). Auditory discrimination learning in zebra finches: effects of sex, early life conditions and stimulus characteristics. Anim Behav.

[CR24] Lachlan RF, van Heijningen CA, Ter Haar SM, ten Cate C (2016). Zebra finch song phonology and syntactical structure across populations and continents—a computational comparison. Front Psychol.

[CR25] Lawson SL, Fishbein AR, Prior NH, Ball GF, Dooling RJ (2018). Relative salience of syllable structure and syllable order in zebra finch song. Anim Cogn.

[CR26] Lehongre K, Aubin T, Robin S, Del Negro C (2008). Individual signature in canary songs: contribution of multiple levels of song structure. Ethology.

[CR27] Lenth RV (2016). Least-squares means: the R Package lsmeans. J Stat Softw.

[CR28] Lipkind D, Marcus GF, Bemis DK, Sasahara K, Jacoby N, Takahasi M, Suzuki K, Feher O, Ravbar P, Okanoya K, Tchernichovski O (2013). Stepwise acquisition of vocal combinatorial capacity in songbirds and human infants. Nature.

[CR29] Lipkind D, Zai AT, Hanuschkin A, Marcus GF, Tchernichovski O, Hahnloser RHR (2017). Songbirds work around computational complexity by learning song vocabulary independently of sequence. Nat Commun.

[CR30] Liu WC, Gardner TJ, Nottebohm F (2004). Juvenile zebra finches can use multiple strategies to learn the same song. Proc Natl Acad Sci.

[CR31] Marler P, Peters S (1987). A sensitive period for song acquisition in the song sparrow, *Melospiza*
*melodia*: a case of age-limited learning. Ethology.

[CR32] Mizuhara T, Okanoya K (2020). Do songbirds hear songs syllable by syllable?. Behav Processes.

[CR33] Mol C, Bolhuis JJ, Moorman S (2021). Vocal learning in songbirds: the role of syllable order in song recognition. Philos Trans R Soc Lond B Biol Sci.

[CR34] Plamondon SL, Rose GJ, Goller F (2010). Roles of syntax information in directing song development in white-crowned sparrows (*Zonotrichia*
*leucophrys*). J Comp Psychol.

[CR35] Riebel K, Slater PJB (1999). Song type switching in the chaffinch, *Fringilla*
*coelebs*: timing or counting?. Anim Behav.

[CR36] Soha JA, Marler P (2001). Vocal syntax development in the white-crowned sparrow (*Zonotrichia*
*leucophrys*). J Comp Psychol.

[CR37] Spierings MJ, ten Cate C (2016). Budgerigars and zebra finches differ in how they generalize in an artificial grammar learning experiment. Proc Natl Acad Sci.

[CR38] van Buuren S, Groothuis-Oudshoorn K (2011). Mice: multivariate imputation by chained equations in R. J Stat Softw.

[CR39] van Heijningen CA, de Visser J, Zuidema W, ten Cate C (2009). Simple rules can explain discrimination of putative recursive syntactic structures by a songbird species. Proc Natl Acad Sci.

[CR40] van Heijningen CA, Chen J, van Laatum I, van der Hulst B, ten Cate C (2013). Rule learning by zebra finches in an artificial grammar learning task: which rule?. Anim Cogn.

[CR41] Vernes SC, Kriengwatana BP, Beeck VC, Fischer J, Tyack PL, ten Cate C, Janik VM (2021). The multi-dimensional nature of vocal learning. Philos Trans R Soc Lond B Biol Sci.

