# Supplementary material for: Zebra finches (Taeniopygia guttata) demonstrate cognitive flexibility in using phonology and sequence of syllables in auditory discrimination
Source: Anim Cogn. 2023 Mar 19;26(4):1161–75. doi: 10.1007/s10071-023-01763-4 (PMC10345033; doi:10.1007/s10071-023-01763-4)
Supplement: Supplementary file 3 — Supplementary file3 (DOCX 18 KB) [file 10071_2023_1763_MOESM3_ESM.docx]

**Table S1 Post hoc test results of Binomial GLMMs for the comparisons of two Jumbled versions in the ‘Same syllables’ training group**

| **Stimuli** | **Training_Group** | **estimate** | **SE** | **z.ratio** | ***p*.value** |
| --- | --- | --- | --- | --- | --- |
| 1. Correct rate of responses ~ Test_Treatment + (1\|Bird_ID) + (1\|Age) + (1\|Number_of_Training_Trials),   Sound A in Same-syllables group | | | | | |
| **MiddleJumbled - Training** | **Same** | **-0.907** | **0.181** | **-5.005** | **1.45e-06** |
| **FullJumbled - Training** | **Same** | **-1.817** | **0.178** | **-10.199** | **< 1e-07** |
| **FullJumbled - MiddleJumbled** | **Same** | **-0.909** | **0.160** | **-5.672** | **< 1e-07** |
| 1. Response rate of trials ~ Test_Treatment + (1\|Bird_ID) + (1\|Age) + (1\|Number_of_Training_Trials),   Sound A in Same-syllables group | | | | | |
| **MiddleJumbled - Training** | **Same** | **-1.388** | **0.281** | **-4.940** | **<1e-05** |
| **FullJumbled - Training** | **Same** | **-1.528** | **0.278** | **-5.490** | **<1e-05** |
| FullJumbled - MiddleJumbled | Same | -0.140 | 0.200 | -0.700 | 0.76 |

Response variables in GLMMs: (a) the proportion of correct responses if birds from the Same-syllables group respond to Sound A; and (b) the proportion of trials that birds from the Same-syllables group respond with pecking A or B. Only information about the two Jumbled versions in the ‘Same syllables’ training group were shown in this table. Bold indicates significance.
